# Surgical Outcomes of Different Deep Anterior Lamellar Keratoplasty Techniques—A Single-Centre UK Study

**DOI:** 10.3390/jcm13133644

**Published:** 2024-06-21

**Authors:** Mohamed Elalfy, Ahmed Negm, Shady Soliman, Hasan Naveed, Samer Hamada, Artemis Matsou, Mansour Hassan, Ahmed Atef, Zisis Gatzioufas, Waleed Mahran

**Affiliations:** 1 Queen Victoria Hospital Foundation Trust, East Grinstead RH19 3DZ, UK; 2 Maidstone and Tunbridge Wells NHS Trust, Maidstone TN24QJ, UK; 3 Research Institute of Ophthalmology, Giza 3724827, Egyptshadiance@yahoo.com (S.S.);; 4 Department of Ophthalmology, Faculty of Medicine, Beni Suef University, Beni Suef 62514, Egyptwaleedmahran@hotmail.com (W.M.); 5 Department of Ophthalmology, University Hospital Basel, 4031 Basel, Switzerland

**Keywords:** DALK, BB-DALK, manual DALK, femtosecond DALK, keratoplasty

## Abstract

**Background:** Anterior lamellar keratoplasty (ALK) is a less invasive procedure than PK, and thus avoids many of the intraocular complications associated with PK. DALK can be performed using several different techniques, with either a manual dissection, a keratome or femtosecond-laser assisted dissection, or with a big bubble technique. To analyse the outcomes and compare the results of three deep anterior lamellar keratoplasty (DALK) techniques. **Methods:** This study included 105 DALK cases performed at Queen Victoria Hospital, East Grinstead, UK, in the period between January 2016 and May 2022. Cases were classified into four groups based on technique: BB-DALK, manual DALK, FS-DALK and ‘converted to PK group’. **Results:** There was significant improvement in VA and Kmax compared to the preoperative values in all groups. There was no significant difference detected in VA and Kmax between all groups. **Conclusions:** Performing DALK surgery with any suitable technique (manual, big-bubble or femtosecond-assisted) is effective and causes significant improvements in VA and Kmax, even in cases where a conversion to penetrating keratoplasty is required. However, every technique has its pros and cons and should be tailored according to surgeon preference and individual case pathology.

## 1. Introduction

Lamellar keratoplasty (LK) is a non-open-sky and less invasive procedure than penetrating keratoplasty (PK), and thus has the advantage of avoiding many of the intraocular complications associated with PK [1]. The fundamental concept behind deep anterior lamellar keratoplasty (DALK) relies on baring the Descemet’s membrane (DM). This can be achieved by either manual dissection or through a big-bubble-assisted planned exposure of the DM. Both techniques can be achieved either with assistance of microkeratome or with a femtosecond (FS) laser [2,3,4]. This is important as the apposition of the donor button in relation to the bare DM provides an interface of high optical quality [4]. 

The superiority of one DALK technique over another has not been established in the literature, and decision making in clinical practice is often based on the surgeon’s preference and the clinical findings. To explore this further, our centre shares its real-world experience of clinical outcomes to compare three different DALK techniques.

## 2. Materials and Methods

This study was approved by the local institutional review board (project ID 653) and adhered to the tenets of the Declaration of Helsinki. All surgical DALK cases performed at Queen Victoria Hospital in the period between January 2016 and May 2022 were included. A total of 105 eyes were included in this study (32 female and 73 male). Selected patients were divided into 4 groups by surgical technique: successful BB DALK, FS DALK, Manual DALK and ‘converted to PK’. 

### 2.1. Groups Defined by Surgical Techniques 

#### 2.1.1. DALK

A partial trephination of the patients’ corneas with a diameter ranging from 7.25 to 8.0 mm was performed with a vacuum Trephine (Moria, France), set at approximately 60% of the thinnest preoperative stromal thickness which was measured by anterior segment optical coherence tomography (AS-OCT) (RTVue, Optovue Inc., Fremont, CA, USA). Then, a 27-gauge needle attached to a 5 mL air-filled syringe which was bent with the bevel facing down was carefully inserted tangentially into the paracentral cornea at an approximate depth of 90% stromal thickness. The needle was advanced into mid-cornea and air was injected into the stroma with the intention of achieving a ‘big bubble’. Paracentesis was subsequently performed followed by manual dissection and excision of the anterior stroma with a crescent blade (2.25 mm, BD Visitec, Warwickshire, UK). Then, air was released by rapidly but carefully incising the remaining posterior stroma (and/or the DM), which was then divided into four quadrants and excised. 

Donor was trephined to be 0.25 mm larger than the recipient bed. After that, removal of the donor endothelium and the DM was carried out. This donor button, devoid of its endothelium and DM, was positioned onto the exposed DM of the recipient bed and sutured in place with 16 interrupted 10-0 nylon sutures with buried knots.

#### 2.1.2. FS DALK

For FS-DALK cases, a pre-programmed mushroom pattern was cut into both donor and host corneas using the Intralase enabled keratoplasty (IEK) tab of the treatment planning software on the Femto LDV Z6 femtosecond laser (Ziemer Ophthalmic Systems AG, Port, Switzerland). 

In the host cornea, maximum depth was set at the OCT measured depth minus 80 µm residual bed, within minimum 6 mm diameter. Diameter of the anterior side cut was set at 8.2 mm for most cases.

In the donor cornea, a reciprocal mushroom cut pattern was programmed with reference to the host cut, setting the anterior side cut diameter to the host diameter plus 0.25 mm and the lamellar ring cut depth at host depth plus 20 µm to allow for donor tissue deturgescence post transplantation. The donor corneal button was mounted on an artificial anterior chamber (Barron artificial anterior chamber, Katena, Denville, NJ, USA) using a thin layer of cohesive OVD to cover the anterior surface of the artificial chamber mount and filtered air to bring the chamber to a firm physiological pressure after the locking ring had been engaged symmetrically over the donor corneal limbus.

#### 2.1.3. Manual DALK

After a localized peritomy at a 1 mm distance in the superior limbus, a 5 mm wide and 350 mm deep incision was made using a diamond knife. Then, a side port incision was made and the anterior chamber was filled with air to help visualise the corneal depth of the dissection. Clear corneal stromal dissection was performed with a crescent blade, and then deep dissection was performed with a special corneal splitter (deep lamellar corneal dissector, 6–607; Duckworth and Kent). The scleral tunnel was sutured with 10-0 nylon, the anterior chamber was partially evacuated of air, and then the corneal pocket was filled with viscoelastic. The cornea was then trephined with a Hessburg–Barron suction trephine, and remaining stromal attachments were cut with curved micro-scissors until the DM was bared. The recipient bed was thoroughly irrigated to remove all viscoelastic and debris. Punching of the donor graft was then performed and the endothelium and DM were removed with a dry cellulose sponge and fine forceps. The donor button was sutured to the recipient stromal bed with 10-0 nylon sutures.

#### 2.1.4. Converted to Penetrating Keratoplasty

This group included patients who were planned as either manual, BB or FS DALK but had to be converted to PK due to large rupture of DM during surgery.

### 2.2. Data Collection

Data were collected by reviewing the case notes and electronic patient records in a customised MS Excel (Version 2405) (Microsoft Corp, Redmond, WA, USA) document. Uncorrected visual acuity (UCVA) and PinHole VA (PHVA) were noted. All VA readings were recorded preoperatively, at 1 week, 1 month and 6 months after suture removal and at the last follow-up visit post-surgery.

Preoperative history notes were also perused to extract information regarding previous ocular surgery, such as corneal collagen cross-linking or intracorneal ring segment implantation. Operative details including donor punch diameter; host trephination diameter, BB result, FS laser depth and diameter and side cut; perforation into the anterior chamber; intraoperative conversion to penetrating keratoplasty; and suturing technique were all noted. Early and late postoperative complications were recorded and included presence of a double anterior chamber, IOP elevation, suture related complications, herpes simpex virus (HSV) recurrence, corneal neovascularization, interface opacification and graft failure or rejection. 

### 2.3. Statistical Analysis

Data were collected, revised, coded and entered into the Statistical Package for Social Science (IBM SPSS) version 23. The quantitative data were presented as mean, standard deviations and ranges. In addition, categorical variables were presented as numbers and percentages. 

The comparison between groups with categorical data were performed by using the Chi-square test. The comparison between two independent groups with quantitative data and parametric distribution were performed by using Independent *t*-tests while with non-parametric distribution were performed by using the Mann–Whitney test. 

The comparison between more than one independent group using quantitative data and non-parametric distribution were performed by using ANOVA or Kruskal–Wallis, where data were not homogenously distributed. 

The confidence interval was set to 95% and the margin of error accepted was set to 5%. So, the following *p*-values were considered to be significant:

- *p* > 0.05: Statistically insignificant
- *p* < 0.05: Statistically significant
- *p* < 0.01: Highly significant.

## 3. Results

We identified 105 consecutive cases of planned DALK performed within the period from January 2016 to May 2022. They were classified into four groups (Table 1): BB group (28 patients), FS group (15 patients), manual DALK group (41 patients) and ‘converted to PK group’ (21 patients). The latter group included patients who started to be treated with the BB DALK or Manual DALK techniques but then had to be converted to PK during surgery following a macroperforation. All patients included were followed up as per the hospital’s DALK follow-up protocol.

### 3.1. Visual Outcome

The mean value of PHVA in the BB-DALK group preoperatively was (0.32 ± 0.25), in the FS-DALK group it was (0.28 ± 0.24), in the manual DALK group it was (0.20 ± 0.20) and in the ‘Converted to PK’ group it was (0.24 ± 0.18), with no significant differences between the four groups (*p* = 0.128). Postoperatively, at 1 week, 1 month, 6 months and at the final follow-up visit, there was a significant improvement in PHVA compared to the preoperative values but with no significant differences between the four groups during any of the follow-up visits (Table 2A,B and Table 3). 

### 3.2. Kmax and Amount of Astigmatism Outcome

The mean value of Kmax in the BB-DALK group preoperatively was (65.82 ± 11.71), in the FS-DALK group it was (64.15 ± 13.30), in the manual DALK group it was (71.55 ± 15.75) and in the ‘Converted to PK’ group was it (72.82 ± 14.35), with no significant differences between the four groups (*p* = 0.111). Postoperatively, at the 1 year follow-up, there was a significant improvement in the Kmax compared to the preoperative values, but with no significant differences noted between the four groups (*p* value). There was no significant improvement in the amount of astigmatism postoperatively compared to preoperative values in all four of the groups (Table 4 and Table 5).

There was a significant elevation in IOP measured by Tonopen between the preoperative and postoperative values in the four groups; in most cases, this was attributed to steroid response and it was controlled by medical treatment with antiglaucoma medications. CCT was significantly improved postoperatively in the four groups compared to the preoperative values.

### 3.3. Intraoperative Complications

DM perforations occurred in a total of forty-one cases. Twenty-of these cases had to be converted to PK due to macroperforation of DM during dissection and all were planned to be either a BB DALK or a manual DALK. One additional case had a DM macrorupture during trephination due to significant corneal thinning. There were another twenty cases, which developed a DM microperforation but were successfully completed as DALK and did not require conversion to PK.

### 3.4. Postoperative Complications

Double anterior chamber occurred in four cases in the BB DALK group and nine cases in the manual DALK group; ff these, only three cases regained DM attachment and cases cases needed rebubbling within the first week. Graft failure occurred in three cases in the BB DALK group, four cases in the FS DALK group, seven cases in the manual DALK groups and in none of the ‘converted to PK’ group cases. Corneal neovascularization occurred in one or two cases in each group except for the manual DALK group in which it occurred in nine cases. Eight of the cases needed another keratoplasty, either PK, DALK, DSAEK or DMEK. IOP elevation occurred in 15 of the total cases and was nearly equal across each group, and most of them were controlled by medical treatment; only two cases needed SST after surgery. HSV recurrence occurred in four cases out of a total of sixteen cases due to post-herpetic scarring. (Table 6).

## 4. Discussion

DALK is currently considered to be the preferred surgical procedure for corneal diseases that do not affect the endothelial layer (e.g., keratoconus, stromal scars and dystrophies), particularly as it carries less risk of complications and produces similar visual acuity outcomes in comparison to full thickness transplantation. There have been several surgical DALK techniques described [5], all of which aim to achieve removal of either the entire, or most of the overlying corneal stroma. Our real-world study evaluating BB-DALK, FS-DALK and manual DALK techniques has critically not found any significant difference between the final outcomes of visual acuity between these DALK techniques to suggest a preference. 

The BB technique for Descemet’s membrane baring, developed by Anwar and Teichmann [6] in 2002, has shown favourable outcomes concerning visual acuity, kera-tometry and astigmatism in patients with keratoconus and superficial corneal scars spar-ing DM. Studies by Fontana et al. [7] and Schiano-Lomoriello et al. [8] have both reported improvements in the final BSCVA in whom big bubble with exposure of the Descemet membrane was achieved. Similarly, other studies also found an improvement in the mean refractive spherical equivalent (MRSE) mean preoperative, mean postoperative and p value of −11.36 ± 2.45, −3.91 ± 1.56 and ≤0.001, respectively. Comparable observations were made by Romano et al. [9], who reported a preoperative MRSE of −11.1 ± 5.6 diopters (D) and an MRSE of −2.6 ± 3.5 D at a postoperative follow-up visit. In our study, the BB DALK group preoperatively showed a mean PHCVA of 0.32 ± 0.25, with a corresponding mean postoperative PHCVA (at final follow-up) of 0.47 ± 0.26 (*p* value 0.000), representing a highly significant improvement in correctable visual acuity.

The advent of femtosecond laser-assisted trephination has been a welcome leap in the field of keratoplasty, allowing more customisation, accuracy and precision. Malyugin et al. [10] (2022) compared manual and laser-cut corneal tunnel creation for intrastromal air injection during an deep anterior lamellar keratoplasty (DALK) and they found out that creating the intrastromal guiding tunnel using FS laser for air injection resulted in a higher rate of BB formation. Further, in 2015, Alio et al. [11] compared the outcomes and healing patterns in FS DALK and manual DALK and they reported that femtosecond assisted and manual DALK show comparable visual and refractive outcomes. 

Buzzonetti et al. [12] performed BB DALK assisted by a femtosecond laser in children and reported that the mean postoperative BCVA was 20/30 (range, 20/25 to 20/30), the mean spherical equivalent was −1.8 ± 1.2 diopters (D) (range, −0.25 to 1.25 D), the mean refractive astigmatism was 1.8 ± 1.4 D (range, 0 to 4.0 D), the mean keratometric astigmatism was 5.1 ± 2.1 D (range, 3.5 to 8.59 D), the mean K value was 46.2 ± 0.8D and the mean corneal thinnest point was 581 ± 46 μm (range, 511–638 μm). Our study’s FS-DALK group showed a mean preoperative PHVA of 0.285 ± 0.244 which improved to a mean postoperative PHVA (at final follow-up) of 0.248 ± 0.193 (*p* value are 0.000), which shows a significant improvement in the mean correctable visual acuity in this group as well.

The refractive outcomes of DALK have been also known to be similarly myopic to those of PK but over a narrower range [5]. This is also supported by our case series, where we observed the average K max in BB DALK group was 64.77 ± 12.35 preoperatively and 55.64 ± 7.18 postoperatively at 1 Y with a significant improvement between both values. We also noted a similar trend in Kmax between the preoperative and postoperative values in the other surgical technique groups. And importantly, there was no significant difference detected when comparing each of the groups with respect to Kmax values. 

### Limitations

This real-world study had inherent limitations due to its retrospective design and the consequent uneven sample size in each of the groups. Whilst all surgeries were performed by different surgeons trained in the DALK procedures, there were subtle differences in their techniques. Further, the study was not powered to detect differences in outcomes for each individual aetiology, and thus the sample size cannot be used to make any conclusions about each pathology. 

## 5. Conclusions

In conclusion, performing DALK with any suitable technique is effective and results in significant improvements in VA and Kmax measurements. Surgeon preference and individual pathological presentation should guide the appropriate selection of a DALK technique.

## Figures and Tables

**Table 1:** Baseline characteristics of patients within each sub-group.

		BB DALK Group		FS DALK Group		Manual DALK Group		Converted Group		Test Value *	*p*-Value	Sig.
		No.	%	No.	%	No.	%	No	%			
Gender	Female	8	28.6%	6	40.0%	14	34.1%	4	19.0%	2.245	0.523	NS
	Male	20	71.4%	9	60.0%	27	65.9%	17	81.0%			
Indication for surgery	KC scar	17	60.7%	3	20.0%	22	53.7%	15	71.4%	10.065	0.018	S
	HSV scar	6	21.4%	2	13.3%	6	14.6%	2	9.5%	1.415	0.702	NS
	LSCD	0	0.0%	0	0.0%	4	9.8%	2	9.5%	4.415	0.220	NS
	Post LVC ectasia	1	3.6%	1	6.7%	0	0.0%	0	0.0%	3.441	0.328	NS
	Post DALK scar	0	0.0%	0	0.0%	1	2.4%	0	0.0%	1.576	0.665	NS
	Stromal scarring after SALK	0	0.0%	2	13.3%	0	0.0%	0	0.0%	12.233	0.007	HS
	Post infection scar	0	0.0%	3	20.0%	3	7.3%	1	4.8%	6.529	0.089	NS
	Superficial Scarring	1	3.6%	0	0.0%	1	2.4%	0	0.0%	1.208	0.751	NS
	Lattice Dyst	0	0.0%	2	13.3%	1	2.4%	0	0.0%	7.398	0.060	NS
	Post PK ectatic graft	0	0.0%	1	6.7%	0	0.0%	1	4.8%	4.018	0.260	NS
	SJS	0	0.0%	0	0.0%	1	2.4%	0	0.0%	1.576	0.665	NS
	Aniridia	0	0.0%	0	0.0%	1	2.4%	0	0.0%	1.576	0.665	NS
	KG	0	0.0%	0	0.0%	1	2.4%	0	0.0%	1.576	0.665	NS
	Schnyder Dystrophy	1	3.6%	1	6.7%	0	0.0%	0	0.0%	3.441	0.328	NS
	Granular Dystrophy	1	3.6%	0	0.0%	0	0.0%	0	0.0%	2.776	0.427	NS
	Reis Buckler Dystrophy	1	3.6%	0	0.0%	0	0.0%	0	0.0%	2.776	0.427	NS

*p*-value > 0.05: Non significant (NS); *p*-value < 0.05: Significant (S); *p*-value < 0.01: highly significant (HS); *: Chi-square test.

**Table 2:** (**A**) Uncorrected visual acuity and pinhole visual acuity measurements of each of the subgroups at pre- and various post-op visits. Comparative analysis between different groups. (**B**) Uncorrected visual acuity and pinhole visual acuity measurements of each of the subgroups at pre- and various post-op visits. Comparative analysis by time periods.

(A)										
Visual Acuity			BB DALK Group	FS DALK Group		Manual DALK Group	Converted Group	Test Value ‡	*p*-Value	Sig.
			No. = 28	No. = 15		No. = 41	No. = 21			
**UCVA**										
Pre		Mean ± SD	0.095 ± 0.115	0.096 ± 0.086		0.055 ± 0.108	0.091 ± 0.127	8.016	0.046	S
		Range	0.001–0.4	0.01–0.32		0.001–0.63	0.01–0.5			
1 week		Mean ± SD	0.075 ± 0.085	0.038 ± 0.048		0.050 ± 0.048	0.081 ± 0.069	9.855	0.020	S
		Range	0.01–0.4	0.01–0.16		0.001–0.16	0.01–0.25			
1 month		Mean ± SD	0.103 ± 0.074	0.035 ± 0.039		0.050 ± 0.050	0.125 ± 0.104	26.336	0.000	HS
		Range	0.01–0.32	0.01–0.125		0.001–0.16	0.01–0.5			
6 month		Mean ± SD	0.110 ± 0.077	0.084 ± 0.089		0.089 ± 0.104	0.116 ± 0.108	5.697	0.127	NS
		Range	0.01–0.32	0.002–0.32		0.001–0.5	0–0.5			
ASR		Mean ± SD	0.141 ± 0.089	0.125 ± 0.110		0.103 ± 0.108	0.144 ± 0.145	5.354	0.148	NS
		Range	0.01–0.4	0.001–0.32		0.001–0.5	0.01–0.63			
Last follow-up		Mean ± SD	0.181 ± 0.118	0.126 ± 0.128		0.107 ± 0.120	0.139 ± 0.098	10.670	0.014	S
		Range	0.02–0.5	0.001–0.5		0.002–0.5	0–0.32			
**PH**										
Pre		Mean ± SD	0.323 ± 0.248	0.285 ± 0.244		0.202 ± 0.201	0.236 ± 0.176	5.677	0.128	NS
		Range	0.001–0.8	0.01–0.8		0.001–0.63	0.02–0.7			
1 week		Mean ± SD	0.184 ± 0.162	0.057 ± 0.065		0.140 ± 0.172	0.179 ± 0.126	11.248	0.010	S
		Range	0.01–0.5	0.01–0.2		0.001–0.8	0.01–0.4			
1 month		Mean ± SD	0.301 ± 0.178	0.075 ± 0.091		0.138 ± 0.134	0.277 ± 0.168	30.832	0.000	HS
		Range	0.016–0.8	0.01–0.32		0.001–0.5	0.03–0.63			
6 month		Mean ± SD	0.311 ± 0.164	0.143 ± 0.119		0.225 ± 0.186	0.241 ± 0.156	11.176	0.011	S
		Range	0.1–0.8	0.002–0.4		0.001–0.63	0–0.5			
ASR		Mean ± SD	0.360 ± 0.207	0.198 ± 0.131		0.276 ± 0.233	0.278 ± 0.212	6.037	0.110	NS
		Range	0.1–0.8	0.016–0.4		0.001–0.8	0.01–0.8			
Last follow-up		Mean ± SD	0.468 ± 0.261	0.248 ± 0.193		0.314 ± 0.224	0.313 ± 0.250	8.480	0.037	S
		Range	0.125–1	0.016–0.63		0.002–0.8	0–0.8			
**(B)**										
**Visual Acuity**		**Pre**	**1 Week**	**1 Month**	**6 Month**	**ASR**	**Last Follow up**	**Test Value #**	***p*** **-Value**	**Sig.**
**UCVA**										
BB DALK	Mean ± SD	0.095 ± 0.115	0.075 ± 0.085	0.103 ± 0.074	0.11 ± 0.077	0.141 ± 0.089	0.181 ± 0.118	33.096	0.000	HS
	Range	0.001–0.4	0.01–0.4	0.01–0.32	0.01–0.32	0.01–0.4	0.02–0.5			
FS DALK	Mean ± SD	0.096 ± 0.086	0.038 ± 0.048	0.035 ± 0.039	0.084 ± 0.089	0.125 ± 0.110	0.126 ± 0.128	11.884	0.036	S
	Range	0.01–0.32	0.01–0.16	0.01–0.125	0.002–0.32	0.001–0.32	0.001–0.5			
Manual DALK	Mean ± SD	0.055 ± 0.108	0.050 ± 0.048	0.050 ± 0.05	0.089 ± 0.104	0.103 ± 0.108	0.107 ± 0.12	36.787	0.000	HS
	Range	0.001–0.63	0.001–0.16	0.001–0.16	0.001–0.5	0.001–0.5	0.002–0.5			
Converted	Mean ± SD	0.091 ± 0.127	0.081 ± 0.069	0.125 ± 0.104	0.116 ± 0.108	0.144 ± 0.145	0.139 ± 0.098	10.838	0.055	NS
	Range	0.01–0.5	0.01–0.25	0.01–0.5	0–0.5	0.01–0.63	0–0.32			
**PH**										
BB DALK	Mean ± SD	0.323 ± 0.248	0.184 ± 0.162	0.301 ± 0.178	0.311 ± 0.164	0.360 ± 0.207	0.468 ± 0.261	37.141	0.000	HS
	Range	0.001–0.8	0.01–0.5	0.016–0.8	0.1–0.8	0.1–0.8	0.125–1			
FS DALK	Mean ± SD	0.285 ± 0.244	0.057 ± 0.065	0.075 ± 0.091	0.143 ± 0.119	0.198 ± 0.131	0.248 ± 0.193	28.323	0.00	HS
	Range	0.01–0.8	0.01–0.2	0.01–0.32	0.002–0.4	0.016–0.4	0.016–0.63			
Manual DALK	Mean ± SD	0.202 ± 0.201	0.140 ± 0.172	0.138 ± 0.134	0.225 ± 0.186	0.276 ± 0.233	0.314 ± 0.224	59.434	0.000	HS
	Range	0.001–0.63	0.001–0.8	0.001–0.5	0.001–0.63	0.001–0.8	0.002–0.8			
Converted	Mean ± SD	0.236 ± 0.176	0.179 ± 0.126	0.277 ± 0.168	0.241 ± 0.156	0.278 ± 0.212	0.313 ± 0.250	10.731	0.057	NS
	Range	0.02–0.7	0.01–0.4	0.03–0.63	0–0.5	0.01–0.8	0–0.8			

*p*-value > 0.05: Non significant (NS); *p*-value < 0.05: Significant (S); *p*-value < 0.01: highly significant (HS); ‡: Kruskal–Wallis test. *p*-value > 0.05: Non significant (NS); *p*-value < 0.05: Significant (S); *p*-value < 0.01: highly significant (HS) #: Friedman test.

**Table 3:** Post Hoc analysis by least significant difference (LSD) of VA measurements between all subgroups.

Visual Acuity	BB DALK vs. FS DALK	BB DALK vs. Manual DALK	Manual DALK vs. FS DALK	Converted vs. BB DALK	Converted vs. FS DALK	Converted vs. Manual DALK
**UCVA**						
Pre	0.682	0.053	0.030	0.775	0.527	0.029
1 week	0.014	0.158	0.198	0.489	**0.006**	0.052
1 month	**0.000**	**0.001**	0.333	0.553	**0.000**	**0.000**
Last follow-up	0.053	**0.001**	0.557	0.266	0.391	0.129
**PH**						
1 week	**0.006**	0.116	0.149	0.839	**0.002**	0.067
1 month	**0.000**	**0.000**	0.121	0.677	**0.000**	**0.001**
6 month	**0.001**	**0.028**	0.175	0.140	0.063	0.591
Last follow-up	**0.009**	**0.018**	0.373	0.076	0.573	0.875

**Table 4:** Pre-operative intraocular pressure and tomography measurements between groups.

PRE		BB DALK Group	FS DALK Group	Manual DALK Group	Converted Group	Test Value	*p*-Value	Sig.
		No. = 28	No. = 15	No. = 41	No. = 21			
IOP	Mean ± SD	15.13 ± 4.14	13.11 ± 4.15	15.05 ± 3.23	12.77 ± 4.19	2.349 •	0.077	NS
	Range	5–22	5–21	10–21	3–20			
K1	Mean ± SD	48.63 ± 10.56	51.57 ± 9.80	57.89 ± 10.06	55.10 ± 13.29	2.056 •	0.113	NS
	Range	35.7–68	37.5–70.2	40.1–70.2	30.2–82.4			
K2	Mean ± SD	53.02 ± 12.43	55.99 ± 10.82	64.18 ± 12.03	60.57 ± 13.27	2.693 •	0.052	NS
	Range	41.7–74.4	40–76	42.6–80	35.9–84.3			
K mean	Mean ± SD	50.83 ± 11.32	53.78 ± 10.16	61.03 ± 10.93	57.84 ± 13.15	2.436 •	0.071	NS
	Range	39.98–71.2	39.25–70.45	41.35–75.1	34.6–83.35			
K max	Mean ± SD	64.15 ± 13.30	64.77 ± 12.35	72.82 ± 14.35	71.55 ± 15.75	2.062 •	0.111	NS
	Range	48.1–87.8	42.5–86.7	45.8–96.6	47.1–101.9			
Astigm amount	Mean ± SD	4.19 ± 4.26	5.43 ± 4.08	6.28 ± 3.97	5.47 ± 3.80	3.821 ‡	0.281	NS
	Range	0.4–13.2	0.3–15.4	0.6–15	0–16.5			
CCT	Mean ± SD	424.30 ± 150.15	418.22 ± 110.52	356.68 ± 92.18	390.11 ± 132.28	1.147 •	0.336	NS
	Range	216–618	265–813	99–514	111–827			

*p*-value > 0.05: Non significant (NS); *p*-value < 0.05: Significant (S); *p*-value < 0.01: highly significant (HS); •: One Way ANOVA test; ‡: Kruskal–Wallis test.

**Table 5:** Post-operative intraocular pressure and tomography measurements between groups.

POST		BB DALK Group	FS DALK Group	Manual DALK Group	Converted Group	Test Value	*p*-Value	Sig.
		No. = 28	No. = 15	No. = 41	No. = 21			
IOP	Mean ± SD	18.39 ± 6.87	16.80 ± 5.33	18.56 ± 7.36	18.81 ± 6.68	0.301 •	0.825	NS
	Range	9–36	8–31	5–36	9–33			
K1	Mean ± SD	41.35 ± 4.42	42.14 ± 2.70	40.94 ± 6.54	40.63 ± 4.41	0.220 •	0.882	NS
	Range	29.2–47.5	39–48.2	24.7–52.6	30.9–47.2			
K2	Mean ± SD	47.70 ± 4.76	47.09 ± 3.87	47.05 ± 5.36	47.44 ± 3.53	0.099 •	0.960	NS
	Range	38.8–55.8	43.1–53.6	34.1–55.3	40.9–52.4			
K mean	Mean ± SD	44.53 ± 4.09	44.61 ± 3.03	43.99 ± 5.43	44.03 ± 3.30	0.104 •	0.957	NS
	Range	35.6–50.55	41.7–50.7	29.4–53.75	36.5–49.05			
K Max post	Mean ± SD	55.64 ± 7.18	54.63 ± 4.66	56.90 ± 5.42	55.55 ± 7.30	0.425 •	0.735	NS
	Range	43.3–72.2	46.7–61.1	48–73.1	45.1–68.5			
Astigm amount	Mean ± SD	5.99 ± 4.19	4.93 ± 2.82	5.77 ± 4.87	6.52 ± 4.01	1.144 ‡	0.766	NS
	Range	0.3–17.4	1.7–8.8	0–23.4	0.6–17.9			
CCT	Mean ± SD	530.68 ± 54.43	502.00 ± 83.27	515.62 ± 70.57	525.68 ± 66.38	0.561 •	0.642	NS
	Range	411–657	374–625	343–633	386–709			

*p*-value >0.05: Non significant (NS); *p*-value <0.05: Significant (S); *p*-value< 0.01: highly significant (HS); •: One Way ANOVA test; ‡: Kruskal Wallis test.

**Table 6:** Post-operative complication data for all groups.

		BB DALK Group		FS DALK Group		Manual DALK Group		Converted Group		Test Value *	*p*-Value	Sig.
		No.	%	No.	%	No.	%	No.	%			
Failure	No	25	89.3%	11	73.3%	34	82.9%	21	100.0%	6.201	0.102	NS
	Yes	3	10.7%	4	26.7%	7	17.1%	0	0.0%			
DMD	No	24	85.7%	14	93.3%	32	78.0%	21	100.0%	6.465	0.091	NS
	Yes	4	14.3%	1	6.7%	9	22.0%	0	0.0%			
IOP elevation	No	24	85.7%	14	93.3%	33	80.5%	19	90.5%	2.015	0.569	NS
	Yes	4	14.3%	1	6.7%	8	19.5%	2	9.5%			
CNV	No	27	96.4%	13	86.7%	32	78.0%	19	90.5%	5.208	0.157	NS
	Yes	1	3.6%	2	13.3%	9	22.0%	2	9.5%			
High astigm	No	26	92.9%	15	100.0%	41	100.0%	19	90.5%	4.938	0.176	NS
	Yes	2	7.1%	0	0.0%	0	0.0%	2	9.5%			
Suture comp	No	27	96.4%	14	93.3%	37	90.2%	20	95.2%	1.182	0.757	NS
	Yes	1	3.6%	1	6.7%	4	9.8%	1	4.8%			
HSV Recurr	No	28	100.0%	14	93.3%	40	97.6%	19	90.5%	3.525	0.318	NS
	Yes	0	0.0%	1	6.7%	1	2.4%	2	9.5%			
Gr dehicence	No	25	89.3%	13	86.7%	34	82.9%	21	100.0%	4.068	0.254	NS
	Yes	3	10.7%	2	13.3%	7	17.1%	0	0.0%			

*p*-value > 0.05: Non significant (NS); *p*-value < 0.05: Significant (S); *p*-value < 0.01: highly significant (HS); *:Chi-square test.

## Data Availability

The authors confirm that data is not freely available due to privacy or ethical restrictions.
